# Moths Behaving like Butterflies. Evolutionary Loss of Long Range Attractant Pheromones in Castniid Moths: A *Paysandisia archon* Model

**DOI:** 10.1371/journal.pone.0029282

**Published:** 2012-01-04

**Authors:** Víctor Sarto i Monteys, Patricia Acín, Glòria Rosell, Carmen Quero, Miquel A. Jiménez, Angel Guerrero

**Affiliations:** 1 Direcció General d'Agricultura i Ramaderia, Generalitat de Catalunya, Barcelona, Catalonia, Spain; 2 Departament de Química Biològica i Modelització Molecular, Institut de Química Avançada de Catalunya-Consejo Superior de Investigaciones Científicas, Barcelona, Catalonia, Spain; 3 Departament de Farmacologia i Química Terapèutica (associate unit to Consejo Superior de Investigaciones Científicas). Universitat de Barcelona. Barcelona, Catalonia, Spain; Ghent University, Belgium

## Abstract

**Background:**

In the course of evolution butterflies and moths developed two different reproductive behaviors. Whereas butterflies rely on visual stimuli for mate location, moths use the ‘female calling plus male seduction’ system, in which females release long-range sex pheromones to attract conspecific males. There are few exceptions from this pattern but in all cases known female moths possess sex pheromone glands which apparently have been lost in female butterflies. In the day-flying moth family Castniidae (“butterfly-moths”), which includes some important crop pests, no pheromones have been found so far.

**Methodology/Principal Findings:**

Using a multidisciplinary approach we described the steps involved in the courtship of *P. archon*, showing that visual cues are the only ones used for mate location; showed that the morphology and fine structure of the antennae of this moth are strikingly similar to those of butterflies, with male sensilla apparently not suited to detect female-released long range pheromones; showed that its females lack pheromone-producing glands, and identified three compounds as putative male sex pheromone (MSP) components of *P. archon*, released from the proximal halves of male forewings and hindwings.

**Conclusions/Significance:**

This study provides evidence for the first time in Lepidoptera that females of a moth do not produce any pheromone to attract males, and that mate location is achieved only visually by patrolling males, which may release a pheromone at short distance, putatively a mixture of Z,E-farnesal, E,E-farnesal, and (E,Z)-2,13-octadecadienol. The outlined behavior, long thought to be unique to butterflies, is likely to be widespread in Castniidae implying a novel, unparalleled butterfly-like reproductive behavior in moths. This will also have practical implications in applied entomology since it signifies that the monitoring/control of castniid pests should not be based on the use of female-produced pheromones, as it is usually done in many moths.

## Introduction

Lepidoptera, including butterflies and moths, are one of the most diverse animal groups with about 160,000 described species [1]. Butterflies (excluding hedylids) are unique in their partner-finding, which is based on visual cues and male-released short-range pheromones; sex pheromone glands have apparently been lost in female butterflies [2]. In contrast, moths use the ‘female calling plus male seduction’ system, in which females release long-range sex pheromones to attract conspecific males; they are generally emitted from epidermal glands in membranous areas in the ovipositor, the ancestral state in ditrysian lepidopterans [3].

Butterflies (skippers and true butterflies) are paraphyletic [4]–[5] and it is likely that such peculiar reproductive behavior evolved independently as an adaptation to diurnal habits. Skippers (Hesperioidea), for instance, are more closely related to hedylids (Hedyloidea) than to true butterflies (Papilionoidea), but hedylids are chiefly nocturnal, and the available data [6] suggest their reproductive behavior is moth-like.

The preponderance of this partner-finding strategy in butterflies naturally leads to the question whether similar behaviors have been evolved in some of the several other lineages of higher Lepidoptera (the ‘moths’) with diurnally active adults. We here report the first such case in *Paysandisia archon* (Lep. Castniidae), a moth recently introduced in Europe from South America which has become a serious pest of palm trees [7].

The Castniidae or “butterfly-moths” form a likely Gondwanan group of brightly coloured, median/large sized moths, occurring in the Neotropics, SE Asia and Australia [8]. They remarkably mimic many butterflies living in the same area in form, colours and habits, forming a truly Batesian mimicry association [9]. They have been placed in the superfamily Sesioidea together with Sesiidae (clearwing moths) and Brachodidae [10], but recent studies have grouped them with some Cossoidea (carpenter moths) in a large, near-monophyletic (but internally unresolved) assemblage that includes Cossoidea, Sesioidea and Zygaenoidea [4]–[5]. Many species in this heterogeneous group are diurnal.

Unique and outstanding in the moth world, castniid males are territorial. They patrol and perch in their habitats, searching or waiting for females -or males or other subjects close to their size-, and fly vigorously after them, in much the same way as butterflies do [7], [9], [11]–[13]. *P. archon* is a powerful flyer, the females actively flying around within their habitat (palm groves where the larvae complete their development to adults) until they are spotted by a patrolling or perching male who chases after them.

Our finding, likely to be widespread in Castniidae, means that a novel butterfly-like reproductive behavior has been found for the first time in moths, revealing the butterfly system, long thought to be unique to butterflies, and based on visual stimuli for mate location and lack of female-released pheromones, has also evolved in lepidopterans other than butterflies. This, in addition to be an authentic evolutionary breakthrough, has important effects on applied entomology since it implies that the monitoring/control of castniid pests should not be based on collecting males by using female-produced pheromones, as it is usually done in many moths. Other methods, like biological control or else, should be considered in this case to avoid spread of castniid pests.

## Materials and Methods

### Ethics Statement

We studied a moth which is not an endangered/protected invertebrate species. All necessary permits were obtained for the described field studies. These were carried out within the premises of the Universitat Autònoma de Barcelona Experimental fields (Barcelona) and in a privately owned plot containing Chusan palms belonging to Mr Eusebi Torrent (Palm Ter Societat Limitada) (Girona).

### Behavioral studies

*P. archon* adults were obtained from field-caught larvae and pupae. The adults were observed in the wild on a 2,400 m^2^ experimental plot close to Girona (Catalonia, Spain), which contained 342 Chusan palm trees (*Trachycarpus fortunei*). Semi-field behavioral assays were performed in two wire mesh cages (1.20×1.50×2.10 m^3^) and one rectangular-shaped mesh tent (1.85×5.00×2.10 m^3^). In such conditions, mating pairs were regularly sighted, although the complex mating behavior observed in the wild, where territoriality and quick and vigorous flights are required, cannot be properly performed unless big areas are available to the moths.

### Stereomicroscopy and flagellomere surface calculations

One/two days after emergence, the adults were killed by freezing at -20°C, and their antennae detached for morphological analysis. Data for flagellomeres number and length were obtained from 20 antennae (10 male and 10 female), whereas 10 antennae (5 male and 5 female) were used for the other variables studied, including flagellomere surface calculations. Antennal flagellomeres were observed under a SZH Olympus stereomicroscope, using a 0.1 mm calibrated ocular micrometer. From each individual we determined the number of flagellomeres per flagellum plus length, width, lateral surface (LS) area, and olfactory sensory surface (OSS) area. To calculate the LS area of the flagellomeres, since all but the terminal one are elliptic cylinders, the formula used was LS = height of the elliptic cylinder x perimeter of its elliptical base. The tiny terminal flagellomere is an elliptic cone, and its LS was calculated accordingly. For the OSS area, we first estimated the ratio between olfactory and non-olfactory (i.e. covered by scales) areas within a particular flagellomere, and then applied such a ratio to the whole LS area of this flagellomere; for flagellomeres located in the basal half of the flagellum, their OSS areas were estimated directly from SEM images using Adobe® Photoshop® CS5 software.

### Scanning electron microscopy

SEM images were used for calculating olfactory sensilla densities within each flagellomere and for measuring sensilla physical features. The same antennae used previously for stereomicroscopy were placed directly on stubs and grounded with carbon paint, before being coated with gold *ca* 50 Å thick by sputtering in an E5000 Sputter Coater (Polaron Equipment Ltd., Watford, UK.). Samples were examined using a Hitachi S-570 (Hitachi Ltd., Tokyo, Japan) electron microscope at 220× to 1,200× magnifications. The OSS of each flagellomere was photographed, and for each sensillar type the sensilla densities were calculated by counting the number of sensilla per area unit. The total number of each olfactory sensillar type per antenna resulted from adding up the numbers of each individual antennal flagellomere. Statistical analysis was carried out using SPSS software (version 15.0 for Windows).

### Electroantennography

The EAG apparatus was commercially available from Syntech. Antennae of *P. archon* males (*n* = 29) and females (*n* = 13) were excised and divided into two halves, but only depolarizations of the distal half were considered, since it is there where most olfactory sensilla are located. The distal half was cut on both ends and fixed to both electrodes with conducting gel. A glass tube (7 cm long×5 mm diameter) with three openings was used as stimulus dispenser. One of these openings was connected to a continuous flow of humidified pure air, the second allowed a complementary air current keep a constant air pressure, and the third one, close to the antenna, housed the stimulus source. Extracts of head, thorax, wings and abdomen of males (*n* = 10), and similar zones plus ovipositor of females (*n* = 12), were obtained by immersion in hexane for 3 h, and the supernatant decanted. The extracts were concentrated to 10 µL and deposited onto a small (2×2 cm) folded Whatman paper which served as the source for the corresponding stimulus inserted in a Pasteur pipette. The antennae were stimulated sequentially with hexane (blank), and three puffs of each stimulus alternatively with the blank. The average of the blank responses was subtracted from that of the stimuli, and the depolarization means were compared for significance using ANOVA followed by two-tailed Student t test, P<0.05.

For GC-EAD analysis, a Focus GC (Thermo Ins.) equipped with a split/splitless injector, a FID detector and a second make-up gas (nitrogen) was used. Helium was the carrier gas and the GC column effluent was split to the EAD preparation in 1∶1 ratio. A HP-5 capillary column (30 m×0.25 mm i.d.×0.25 µm) was used under the following GC conditions: injection at 60°C for 1 min, program at 5°C/min up to 120°C, kept at this temperature for 1 min, program at 10°C/min up to 250°C, and kept at this temperature for 10 min. The extracts were concentrated to 1–2 µL and injected in splitless mode. Porapak volatiles were obtained by aeration of males and females placed individually or in one male∶female pair into 1 L Erlenmeyer flasks containing a rolling wire to facilitate hanging of the insect. The airflow (500 mL/min) was maintained for 48 h at 24±2°C under regular L∶D regime. A total of 4 aerations of a pair male∶female, 4 of only females and 6 of only males were implemented. Volatiles were absorbed on Porapak Q cartridges, extracted with hexane and concentrated to 100 µL.

### Chemical analyses

Volatiles eluted from Porapak cartridges and body extracts were analyzed on a Fisons GC 8000 series coupled to a MD 800 mass spectrometer (Thermo Fisher Sci.) using helium as carrier gas. A HP-5 capillary column (Supelco) (30 m×0.25 mm i.d.×0.25 µm) was used under the following chromatographic conditions: injection at 60°C for 1 min, program of 5°C/min up to 120°C, kept at this temperature for 1 min, program of 10°C/min up to 250°C which was maintained for 10 min. Electron impact (EI) mass spectra were recorded in a m/z 40–400 range. The extracts were carefully concentrated to 1–2 µL and injected in splitless mode. Chemical ionization (CI) mass spectra were recorded in a Agilent 5973 Network MSD coupled to a Agilent GC 6890 Series using methane as the reagent gas. The GC column and the chromatographic conditions were identical as for EI mass spectra.

## Results and Discussion

### 1. Butterfly-like reproductive behavior of castniids

#### The courtship of *P. archon*

It is made up of six consecutive steps. They were singled out through the observation of four successful courtships. The steps that follow extend and complement those previously reported [7].

**Figure 1:**
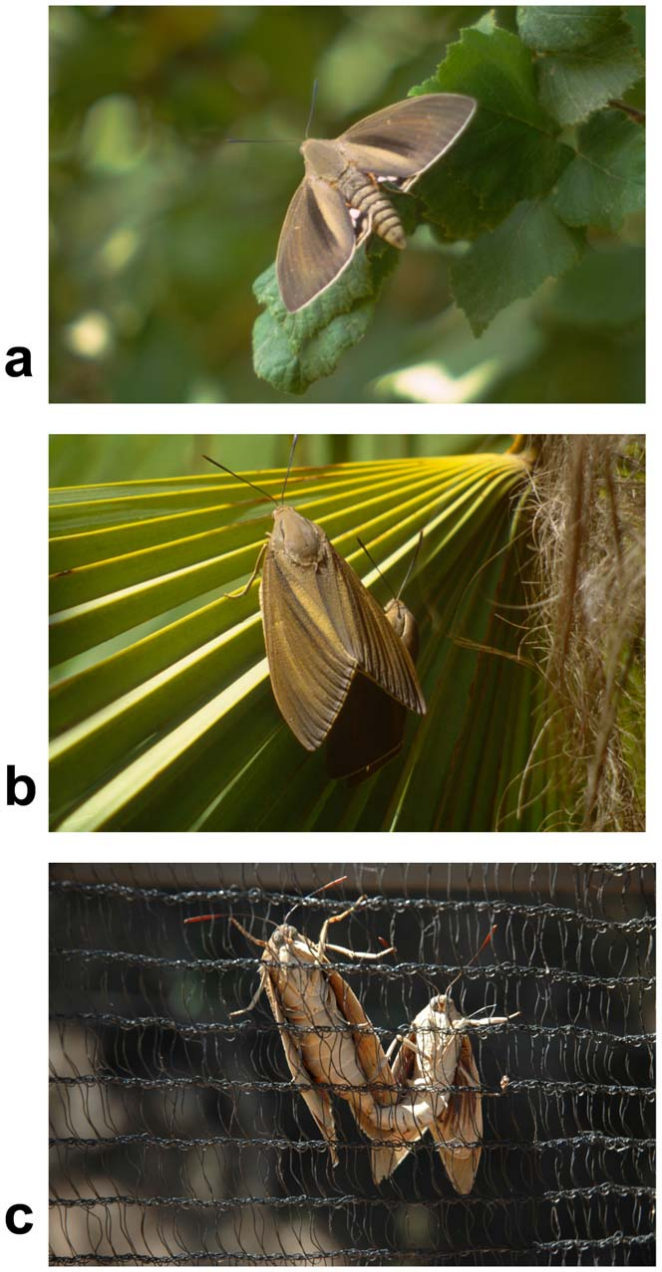
*Paysandisia archon* courtship: **a**. A perching male of *P. archon* about to take off for an intruder (Courtship step 1); **b**. Male orienting himself and approaching the female from below, just after the alighting of both specimens (Courtship step 3); **c**. Pair in copula. The male, on the right, keeps his abdomen bent and attached to that of the female (Courtship step 6).

**1.** Localization/pursuit. The territorial male, who is perching (Fig. 1a) or patrolling, first locates and approaches an intruder, energically flying toward it (*ca.* 20 m/sec). If the latter is a conspecific female, the pursuit that follows is slower than that resulting from the intrusion of a conspecific male. The pair flies together along the palm rows, close to each other (about 10–15 cm), at heights very close to the palm crowns (1.5–2.0 m in the experimental plot). During this phase, apart from visual stimuli, it is likely the female (and also a conspecific male intruder when encountered) receives the presumptive male sex pheromone at close range.

**2.** Alighting. Then, the pair alights, led by the female, on upright surfaces (a palm leaf or crown, the sides of a mesh tent), facing up. The female may walk shortly until reaching a spot where she can rest comfortably, folding her wings in the common noctuoid position, and staying still for the rest of the courtship.

**3.** Orientation. The male, who alighted a few centimeters below the female and has been closely following her movements, also has his wings folded, and moves up toward her, either on the female's right or left side, at a small angle (40–50°) with her (Fig. 1b). There is no male flickering (i.e. a rapid sequence of opening and closing wings).

**4.** Thrusting. While approaching the female from below, the male touches the edges of her wings with his head/antennae, sometimes briefly inserting them under her wings. Also, his antennae and/or legs may also make contact with the side of the female. During this phase both sexes keep their wings fully closed.

**5.** Attempting. While approaching the male curls his abdomen and opens his clasping genital valvae in order to contact and grasp the female copulatory orifice to accomplish the copula.

**6.** Copulation. While in copula both sexes stay motionless, facing up, side by side, the male obviously lower than the female. This copula position (Fig. 1c) is not the typical of Lepidoptera, since generally, after accomplishing the copula, the sexes position themselves on a straight line with their heads at opposite ends. The copulations observed lasted from 24 to 45 min, averaging 37.9 min (n = 4).

#### Sex pheromone glands

Obviously, vision plays a determinant role in castniid reproductive behavior, yet this has been presumed to be moth-like, i.e. mediated by long-range sex pheromones produced and released by females to attract conspecific males [14]–[15]. As we demonstrate herein, this is not the case in *P. archon*.

**Figure 2:**
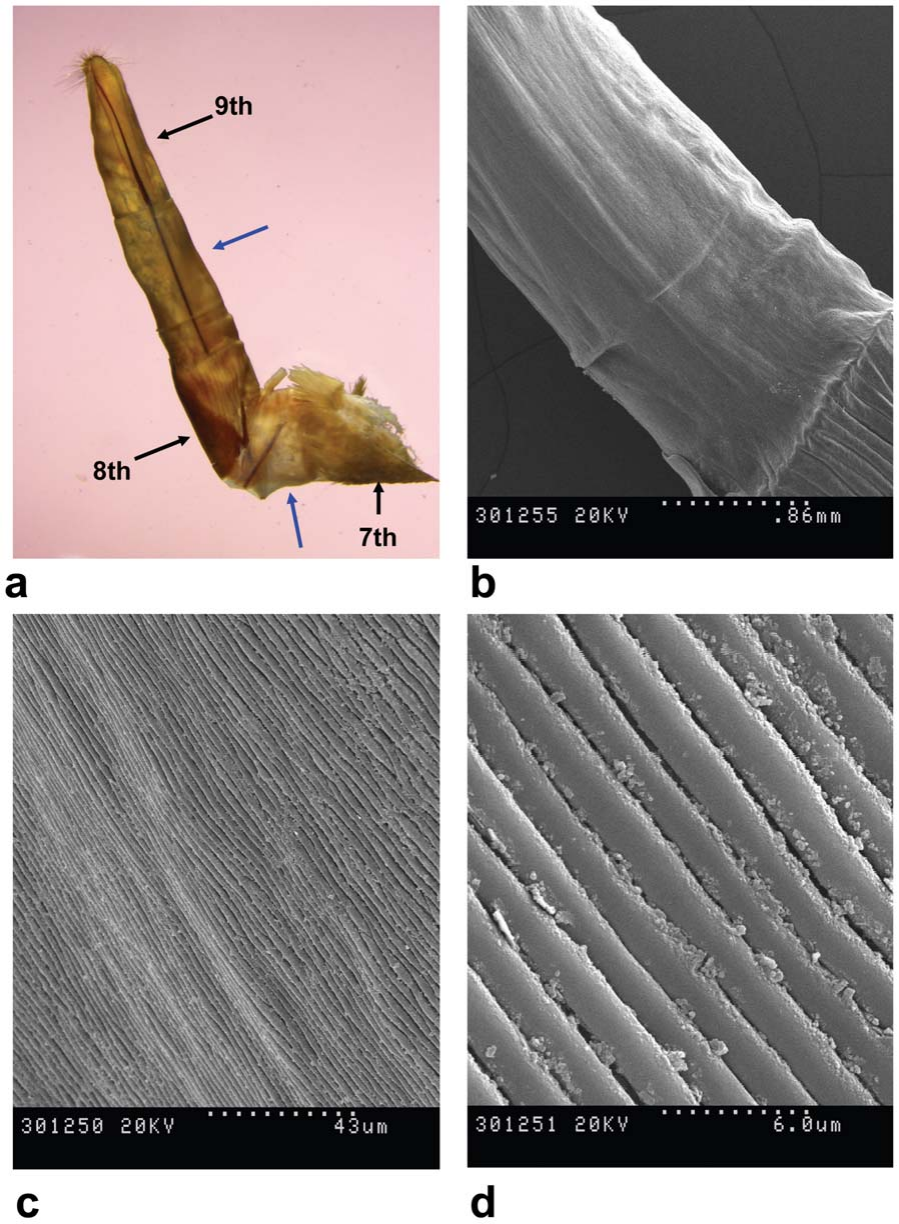
*Paysandisia archon* everted ovipositor and 8th/9th abdominal intersegmental membrane. **a**, side view of fully everted ovipositor (×7.5); black arrows show from top to bottom the 9th, 8th and 7th abdominal segments; blue arrows show the intersegmental membranes between segments 9th-8th (top) and 8th-7th (bottom). **b**, 8th-9th intersegmental membrane showing what looks like a smooth surface (×35). **c**, idem, closeup at 700 magnifications showing multiple longitudinal smooth folds, allowing for extra ovipositor expansion. **d**, idem, closeup at 5000 magnifications.

In most female moths sex pheromone glands occur between the 8^th^ and 9^th^ abdominal segment; when adopting the ‘calling’ position this glandular area is exposed and the pheromone is released [16], [3]. In most Sesioidea and Cossoidea these segments form a telescopic ovipositor. Within these groups and as far as we know, the sex pheromone glands have been specifically sought only in *Synanthedon tipuliformis* and *Paranthrene tabaniformis*; they were located below the intersegmental cuticle between the 8^th^ and 9^th^ abdominal segments, thus conforming to the basic pattern. Such cuticle showed many buds, each topped with one thin and curved spinelike process, supposed to help release the pheromone [17]. However, scanning electron microscopy (SEM) carried out on *P. archon* ovipositors showed that the 8–9 intersegmental cuticle was devoid of such structures, and, instead, multiple longitudinal smooth folds could be seen, simply allowing for ovipositor expansion (Fig. 2). In addition, careful extraction of ovipositors and other female body parts yielded no compounds with putative pheromone activity (see below). In conclusion, it appears that *P. archon* females do not possess any abdominal gland to release a volatile pheromone to attract conspecific males –and this is likely to be widespread in Castniidae.

### 2. Male antennae unsuited to detect long range pheromones

**Figure 3:**
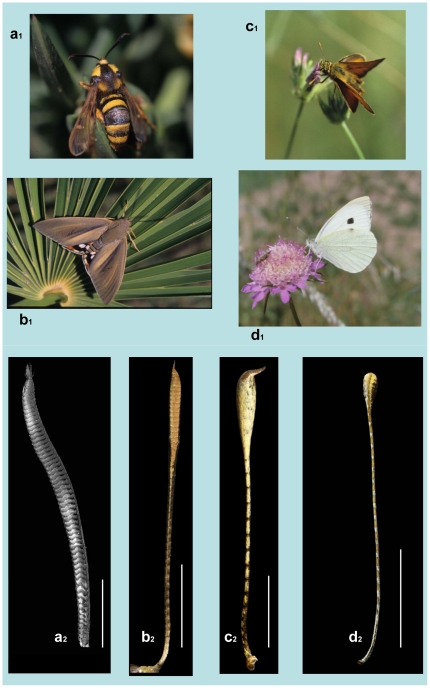
Butterflies, sesiid and castniid moths: **a_1_**, the hornet clearwing *Sesia apiformis* (a sesiid), **b_1_**, the palm borer *Paysandisia archon* (a castniid), and two butterflies: **c_1_**, the large skipper, *Ochlodes venatus*, and **d_1_**, the large white, *Pieris brassicae*, with their respective male antennae, **a_2_**, **b_2_**, **c_2_**, **d_2_**, seen ventrally. Antennal pectinations are only present in the sesiid male, where the olfactory sensory surface (OSS) covers all the ventral side of the antenna. In castniids and butterflies the club forms rather distally and the OSSs are greatly reduced with a clear tendency to concentrate on the club. Scale bars: 2 mm for **a_2_**, **c_2_**; 5 mm for **b_2_**, **d_2_**.

The antennae are the ‘noses’ of moths and butterflies and their morphology and sensilla are suited to their needs [18]–[19]. In moths the antennae generally are sexually dimorphic, with male antennae carrying a population of sensilla devoted to detection of the sex pheromone released by conspecific females. In butterflies, which use sex pheromones only for close-range communication and therefore lack the highly sensitive detection system found in male moths, the antennae are thin and clubbed and show no sexual dimorphism. Castniids and butterflies are day-flying and their antennae are strikingly similar in their looks, with no apparent sexual dimorphism macroscopically. Few other day-flying moths, such as the Sesiinae clearwing moths and the Zygaeninae burnet moths, have also distally thickened antennae, but sexual dimorphism is yet very clear (Fig. 3).

**Figure 4:**
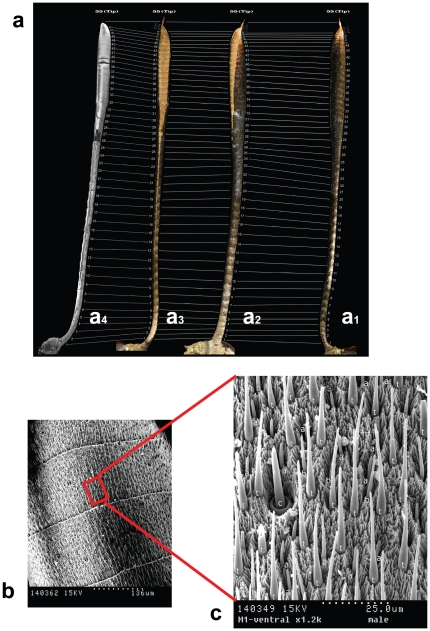
Fine structure of the antenna of *P. archon*. **a**, Image composition of the same antenna of a *P. archon* male showing its flagellomeres: **a_1_**, **a_2_**, **a_3_**, dorsal, lateral and ventral views, respectively, **a_4_** same as **a_3_** in SEM. The olfactory sensory surface (OSS) of each flagellomere (the orange-coloured areas on the club) was measured combining stereomicroscope and SEM images. Sensilla densities along the antenna were obtained by combining each individual flagellomere's OSS with number of sensilla in SEM. **b**, Ventral surface of club flagellomeres showing their OSSs. **c**, Close-up view of a small area of OSS showing three different olfactory sensillar types arising from the mantle of microtricha: (t) sensilla trichodea, (b) s. basiconica, (a) s. auricillica. One stout chaetic sensillum (c) can also be seen.

Since butterflies are more dependent on visual stimuli, and hence their antennae are less important as sensory organs, their number of sensilla, sensillar types, and olfactory sensory surfaces (OSSs) have been dramatically reduced. Therefore, sensilla occur only in very small areas or ‘sensillar fields’ of the antennae, and such an arrangement can be correlated with systematics [20]. In moths, olfactory sensilla cover most of the ventral surface of virtually all flagellomeres, but not in *P. archon* where 85–89% of all OSSs and similar percentages of the olfactory sensilla concentrate on the club. This is closer to butterflies, where most olfactory sensilla are located on the club too. In *P. archon*, the antennal flagellum shows numerous subdivisions or flagellomeres, 54–65 in males (mean ± s.d. = 57.56±3.28, *n* = 9) and 55–63 in females (mean ± s.d. = 58.56±3.13, *n* = 9). They are covered with scales except for the OSS, which is located on their ventral side and bears only four types of sensilla –chaetica, trichodea, basiconica and auricillica–, the latter three being the olfactory sensilla proper (Fig. 4). The OSS begins at flagellomeres 10 to 15 and takes up a narrow ventral middle line, and from there it expands progressively along the sequence of subsequent flagellomeres sideways towards the dorsum. At the club it reaches its maximum cover. In *P. archon* the ratio OSS/total antennal surface is 13–21%, notably lower than that of other moths (>40%), and closer to butterflies where OSSs are greatly reduced (*ca.* 2.8% in the large skipper, *Ochlodes venatus*, and *ca.* 1.7% in the clouded yellow, *Colias crocea*). In addition, *P. archon* females show OSSs *ca.* 6% higher than those of males (T = −5.747, P = 0.005), regardless of antennal length and whole antennal surface, in contrast to most moths where males show generally higher OSSs than females.

Unlike s. basiconica and s. auricillica, s. trichodea are often sexually dimorphic in moths, being generally longer in males than in females [19], [21]. For instance, in the hornet clearwing *Sesia apiformis* these values are (mean ± s.d.) 63.68±9.43 µm for males and 41.14±4.44 µm for females, *n* = 15 for each sex. In butterflies, s. trichodea lengths are more alike, e.g. (mean ± s.d.) 22.84±1.61 µm vs 16.81±1.32 µm in the large skipper and about the same in both sexes 14.59±0.88 µm vs 14.56±0.92 µm in the clouded yellow (male vs female, *n* = 15 for each sex). In *P. archon* though, s. trichodea are somewhat longer in females (mean ± s.d. = 39.15±4.68 µm) than in males (37.51±3.58 µm) (*n* = 200 for each sex, measured from 40 trichodea/specimen, 5 males and 5 females) but significant (T = −3.934, P = 0.000). This is unusual in a moth, and suggests that *P. archon* male antennae might not be suited to detect female-released long range pheromones.

**Figure 5:**
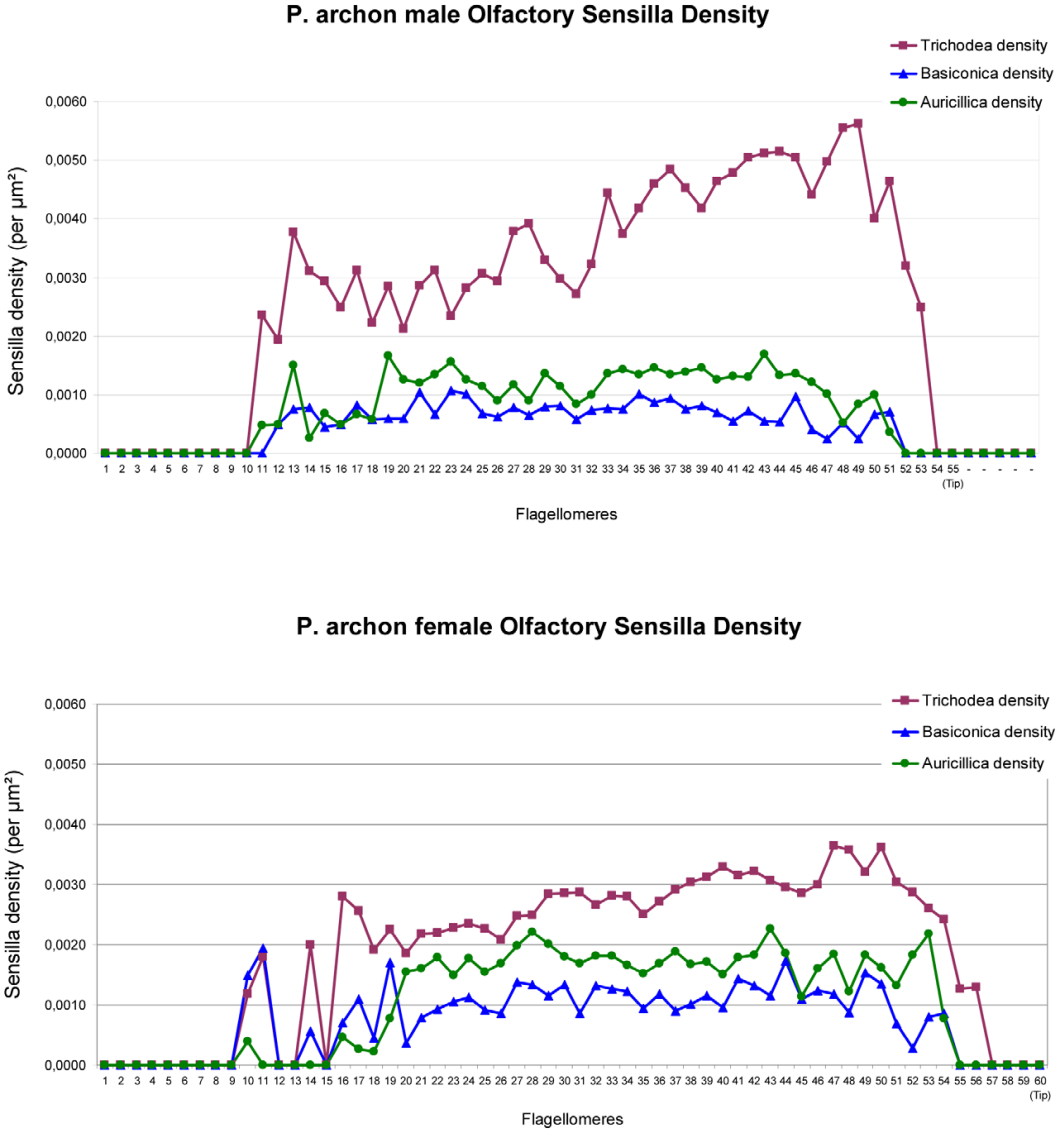
Olfactory sensilla densities along *P. archon* antennal flagellomeres. Sensilla trichodea densities are clearly higher in males (top) than in females (bottom), while the opposite occurs with s. basiconica and s. auricillica. All densities were measured at the flagellomeres' ventral centre strip. Graphs shown are specifically for specimens coded M10 (male) and F9 (female).

With regard to sensilla densities, interestingly, those of s. trichodea of *P. archon* are not uniform along the flagellum but increase upwards towards the tip in both sexes, with male densities being higher than those of females. S. basiconica and auricillica densities are lower than s. trichodea densities in either sex and higher in females than in males (Fig. 5). This implies that trichodea/basiconica and trichodea/auricillica ratios are higher in males, a widespread pattern in moths [19], [21].

The total number of sensilla per antenna, calculated for two *P. archon* males and females, results from combining sensillar densities with OSSs for each and all flagellomeres. The male average (mean ± s.d.) was 15,944±2,164 trichodea (69.50%), 2,473±395 basiconica (10.90%) and 4,474±10 auricillica (19.60%), and for females 19,822±533 trichodea (45.90%), 7,041±492 basiconica (16.31%) and 16,377±2,611 auricillica (37.79%). It is noteworthy that although the density of sensilla trichodea in males is higher than that in females, the total number of sensilla trichodea is higher in females. This is because *P. archon* females posses bigger OSSs than males thereby compensating for s. trichodea lower densities. Such a situation is very unusual in a moth, since in the more widespread scenario s. trichodea are the ones involved in perceiving the pheromones released by females, and therefore they are nearly always more abundant in males [21].

In addition, comparison of 2-DE gel images of the odorant binding protein zone of *P. archon* antennae with that of other lepidopteran species showed a high similarity to that of butterflies and dissimilarity to that of moths (Acín et al. in preparation).

In sum, based on the fine structure of the antennae and in contrast with other moths than have been studied, males *P. archon* appear not to have more highly olfactory capacity than females, particularly for detection of long-range pheromones released by females.

### 3. EAG and behavioral assays

**Figure 6:**
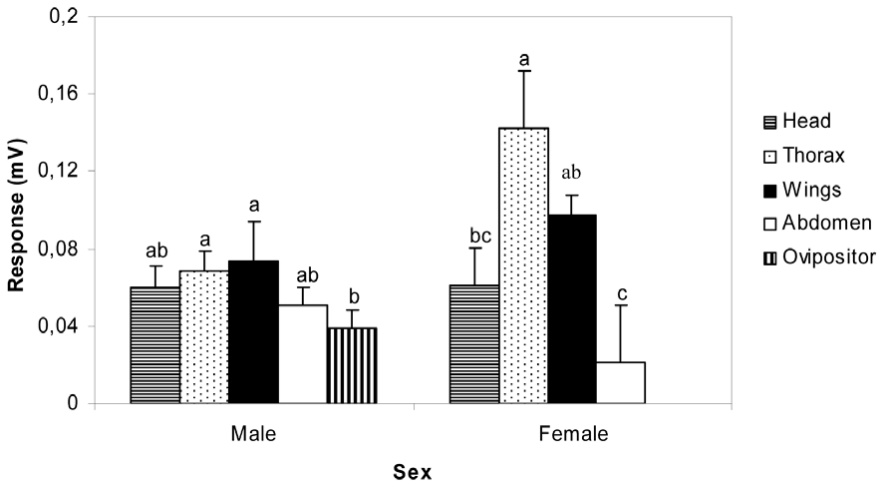
Electrophysiological responses of *P. archon* antennae. EAG response of extracts of different body parts of *P. archon* males and females on antennae (*n* = 9–30) of the opposite sex. Different letters atop bars (± s.e.m.) indicate significant differences among treatments within a sex (ANOVA, two tailed Student t test P<0.05).

Chemical analysis and behavioral assays of sex pheromones have been implemented in many species of moths, but to date only five species of butterflies (two pierids and three nymphalids) have been partially analysed [22]. Because the sites of production/release of pheromones used for intraspecific recognition are not yet known in any castniid, hexane extracts of head, thorax, wings and abdomen from both sexes, and ovipositor from females, were obtained and tested for electrophysiological responses on the opposite sex antennae. For the tests, only distal halves of the antennae bearing ca. 90% of the antennal olfactory sensilla were used. Male and female antennae displayed very low (<0.2 mV) EAG responses to all extracts, the male responses being generally lower than those of females (Fig. 6). Ovipositor extracts elicited the lowest depolarizations in male antennae, whereas male thorax and wing extracts were the most active in antennae of both sexes. Similarly, very low EAG responses (<0.1 mV) were obtained with the pierid butterfly *Pieris brassicae*, when antennae of both sexes were stimulated with extracts of thorax, wings and abdomen.

Porapak-entrapped volatiles from males and females, presented separately and mixed, again elicited scant EAG responses in antennae of the opposite sex (<0.1 mV), the response of females being slightly higher than that of males. In this regard, the pheromone components released by male wings of the nymphalid butterfly *Bicyclus anynana* triggered similar depolarization responses in female antennae (ca. 0.12 mV) (22). These results are consistent with the fact that day-flying butterflies rely on visual stimuli rather than on olfactory cues, the pheromones being generally released by males at close range during courtship [18].

**Figure 7:**
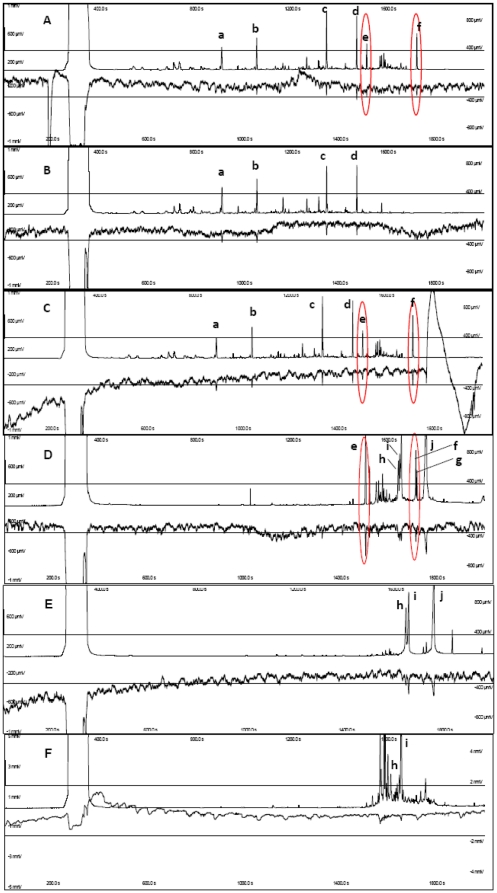
GC-EAD responses of *P. archon* female antennae. Responses to **A**, volatiles of males and females; **B**, female volatiles; **C**, male volatiles; **D**, male wings extract; **E**, male thorax extract; **F**, male abdomen extract. Compounds identification: **a** = nonanal, **b** = decanal, **c** = tetradecamethyl cycloheptasiloxane, **d** = hexadecamethyl cyclooctasiloxane, **e** = *Z,E*-farnesal, **f** = (*E,Z*)-2,13-octadecadienol, **g** = *n*-henicosane; **h** = (*Z*)-11-hexadecenoic acid; **i** = palmitic acid; **j** = oleic acid. Upper trace: FID response. Lower trace: EAD response. Red ellipses highlight *Z,E*-farnesal and (*E,Z*)-2,13-octadecadienol, two components of the putative male sex pheromone.

To identify the components of a putative male pheromone, GC-EAD analyses of male and female volatiles showed a supposedly active response on female antennae by a number of compounds **a**–**f** (Fig. 7, A–C). Among them, compounds **a–d** were present in female volatiles and males and females together but compounds **e–f** were only present in males. Whereas the former chemicals resulted to be artifacts from the Porapak Q used in the volatiles entrainment experiments, compounds **e–f** were identified as (*Z,E*)-3,7,11-trimethyl-2,6,10-dodecatrienal (*Z,E*-farnesal) and (*E,Z*)-2,13-octadecadienol (dienol from now onwards), respectively, by their mass spectra (EI and CI) and retention times compared to authentic samples. These two compounds were also present in male wing extracts (Fig. 7, D) (see below) but not in extracts of male thorax and abdomen (Fig. 7, E, F). In addition, the male wing extracts contained (Z)-11-hexadecenoic acid (**h** in Fig. 7, D), palmitic acid (**i** in Fig. 7, D), and oleic acid (**j** in Fig. 7, D) as EAG active compounds. These chemicals were also present in male thorax and abdomen (no oleic acid here) (Fig. 7, E, F) and in female ovipositors. The ubiquitous presence of these fatty acids in extracts of both males and females and their absence in volatiles preclude any role in the pheromonal communication of this moth. Similar fatty acids were identified in ovipositor extracts of *Castnia licus*, another castniid moth, but their possible activity was not determined [14].

When male (*n* = 3) forewings and hindwings were cut into three strips, basal (A), median (B) and distal (C), the corresponding extracts showed, in addition to *Z,E*-farnesal and dienol, the presence of *E,E*-farnesal (its retention time and mass spectrum matched those of this isomer in a commercial sample). Neither the *Z,E*-, *E,E*-farnesals nor the dienol were detected in female wing extracts. The total amount of *Z,E*-farnesal per insect was generally higher than that of the *E,E* isomer (ca. 1,300 vs 750 ng in the forewings, 1,000 vs 500 ng in the hindwings), both compounds being preferentially detected on the A and B strips. The amount of dienol was similar to that of *E,E-*farnesal and appeared to dramatically decrease with male age (from ca. 750 ng in 7–10-day old individuals to 50 ng in 20-day old specimens). There was no clear difference between the forewing and hindwing contents of the three putative pheromone components.

It is known that males from several lepidopteran families, either moths (e.g. Arctiinae-Noctuidae) or butterflies (Danainae and Ithomiinae-Nymphalidae), accumulate substances from the host plant at the larval stage as a defence mechanism against predators [23], most of which can be subsequently used as pheromone precursors [24]–[25]. Farnesals are generally present in Orchidaceae and Cactaceae, but have not yet been identified in palm trees (Arecaceae), the only host plants known to *P. archon*. These plants contain *E,E*-farnesene and *Z,E*-farnesene from which the corresponding alcohols could be biosynthesized, and thus become precursors of the farnesals released by *P. archon* males. *Z,E*-farnesal and *E,E*-farnesal have also been reported as the male sex pheromone of the pyralid rice moth, *Corcyra cephalonica* [26], where these chemicals are released by glands on the wings and attract females at close range. The dienol found in the presumptive male pheromone of *P. archon* is a common component of pheromones of female sesiid moths [27] to which castniids are related. For behavioral testing, six virgin females were released into a large mesh cage, and adult-size male dummies, made up of cardboard, previously treated with the putative pheromone components, were waived to them. Females simply remained quiet or walked shortly upwards, occasionally pumping in and out the ovipositor tip. When real males were placed just below virgin females which had alighted after having flown together with these males in the cage, successful matings were obtained in 40% (3 out of 8) of the cases. Since *P. archon* courting males do not fan or flick their wings once they have alighted, keeping them folded, an active conveyance of a male sex pheromone toward the female antennae is unlikely during this phase of the courtship. Therefore, the behavioral data available suggest that in this castniid the putative male sex pheromone (MSP) acts and is released in flight only, likely a passive physical process similar to that of the green-veined white butterfly *Pieris napi* [28]. This would help to induce the alighting and stillness of the female, apparently the two signs of acceptance behavior in female castniids. During the rest of the courtship, visual and probably tactile cues are possibly enough for a receptive female to accept the courting male.

### 4. Overview and future prospects

This study provides an integrative analysis of the reproductive behavior of a castniid moth, which shows substantial differences with that known in other moths, but it is strikingly similar to that shown in butterflies. Using a multidisciplinary approach, we have: a) described the steps involved in the courtship of this moth, showing –as in butterflies- that visual cues are the only ones used for mate location, b) showed that the morphology and fine structure of the antennae of this moth are strikingly similar to that of butterflies, with male sensilla apparently not suited to detect female-released long range pheromones, c) showed that *P. archon* females lack pheromone-producing glands, and d) identified three compounds as putative male sex pheromone components of *P. archon*, released from the proximal halves of male forewings and hindwings. In addition, we have hypothesized that the conveyance of the putative male sex pheromone from males to females is probably a passive process associated to the flight phase of the courtship, inducing the female to alight. All the above points together point out the absence in castniids of a long range pheromone released by females to attract males, suggesting a novel, so far undescribed, type of reproductive behavior in moths which has many similarities with that of butterflies.

It should be noted that in some species of burnet moths (Zygaeninae), which like castniids are exclusive day fliers and bear clubbed antennae, two different mate-locating systems have been observed. The females have typical moth-like sex pheromone glands that release the pheromone for attracting males in late afternoon. In the morning females do not release pheromones and can be found by male optical cues exclusively [29]–[30]. Female castniids might have gone a step further by losing their pheromone glands, becoming active fliers to make themselves visible to the territorial patrolling/perching males, and relying solely on visual stimuli to attract conspecific males. The obvious next question that arises from our findings is: what about the other numerous day-flying ditrysian moths (chalcosiine zygaenids, dioptine notodontids, arctiine and agaristine noctuids, etc.) in which similar phenomena could also be found? And, is the clubbed antenna a compulsory condition to this phenomenon in diurnal moths? Such questions should prompt further potentially fruitful inquiries into the behavioral patterns in other diurnal Lepidoptera.
